# The Homœopathic Treatment of Alcoholism

**Published:** 1890-07

**Authors:** 


### BOOK NOTICES.

THE HOMŒOPATHIC TREATMENT OF ALCOHOLISM. By Dr. Gallavardin, of Lyons, France. Translated from the French by Irenæus D. Foulon, A. M., M. D., LL. B., Philadelphia. Hahnemann Publishing House, 921 Arch Street. 1890.

This clever and interesting little book of 138 pages is a plea for genuine homœopathic treatment of drunkenness. The author says:

"A few drunkards can be cured by means of moral instruction, care in diet and hygiene, but in the far larger number, the tendency to inebriety is the result of a species of morbid impulse which is well-nigh irresistible."

"Hitherto homœopathic medicine has proved itself quite as unable to cure drunkenness, because, with rare exceptions, homœopathic physicians, not knowing how to utilize the wealth of their materia medica, have failed to follow these two precepts of their master Hahnemann :

"1st. In the choice of remedies note the intellectual and moral symptoms presented by the patient and produced by the drug proved upon the healthy subject.

"2d. In chronic diseases give in one dose the remedy selected, then let it act for weeks and months.

"Having followed on these two points, the precepts of Hahnemann, I have been able to cure inebriates of their vice in one-half of my cases, when the vice was not hereditary and that by causing to be administered to them, without their knowledge, in their food or their drink, the remedy selected for each of them."

Then follow indications for fourteen remedies useful in drunkenness. These remedies in the order of their importance are as follow: Nux-vomica, Lachesis, Causticum, Sulphur, Calcarea-carbonica, Hepar, Arsenicum-album, Mercurius, Petroleum, Opium, Staphysagria, Conium, Pulsatilla, Magnesia-carbonica. They are to be administered according to indications preferably in the two hundredth potency, one single dose for two, three, four, six, or seven weeks.

From this statement it will be seen that the book teaches sound homœopathic doctrine, and is to be commended to the whole profession. W. M. J.

